# Supplementary material for: Chromothripsis during telomere crisis is independent of NHEJ, and consistent with a replicative origin
Source: Genome Res. 2019 May;29(5):737–49. doi: 10.1101/gr.240705.118 (PMC6499312; doi:10.1101/gr.240705.118)
Supplement: Supplemental Material [file supp_gr.240705.118_Supplemental_file_1.zip › contigs/annotated_contigs/DB56/contig.2.DB56_length_736_mean_cov_7.39402173913.docx]

**DB56_length_736_mean_cov_7.39402173913**

```
ACCCTGCTTCCATTTCTTAACAAAACGTTTTGAAAAGGCAATTATTTAAGGATCTGTTTCTAATAAAGCTTCCGATGTGGCCATGATGT
 >chr7:139566759-139567070 + E=3e-175
TTAATGGAATTGCTGGCAAAGCCCATGAGGGCATGCGTGTTGAAACCATAGAGTAACCATGGCAAAGGGCCTCTCTTCTCCAATGTGCA

GGACACTGGGTTACCTGGCACCGCTGGGCTTCACCTGTTCTTGGAGCATCACAAGTTTTTACCCCTGGAGAAGCGCTATTTGCCAAGGG

AATCTTTCACCATTTCAAATACATCCACTGAAGTTTTTGAAAGC|CACCTGTAATCCCAACACTTTGGAAGGACAGGACTGCAGGACTG
                                              >chr7:137950987-137951396 + E=1e-206
CTTGAAAACAGGAGTTTGAGACCAGCCTGGGCAACATAATGAGACCTCGTTTCTGCAAAAAAAAAAAAAAAATTAGCCAGGCATGGTTG

CGCGCACACCTGTGGTCCCAGTTACCTAGGAGGCTGAGGTGGGAGGATTGCTTCAGCCCAGGAGTTTGAGGCTGCAGTGAGCTGTGAGC

CACTGCACTCCAGCCTGGGTGACAGAGTGAGGCCCTGTCTCCAAAAAAAAAAAAAGTATGACTGTAGATTTTTTTACAGATATATTCCT

TTAACAGAATAAGAATGGCCTGGTTTGTTAAGTGTTTTTTGTTTTGTTTTGTTTTTTTTGAGACAGAGTCTTGCGCTGTCTCC|CTGGC

TGGATTGCACTTGCGCGATCTCCTCG
```
